# Avaliação Crítica do Manejo da Angina Instável em Pronto-Socorro Terciário de Cardiologia

**DOI:** 10.36660/abc.20230049

**Published:** 2024-03-21

**Authors:** Mateus Arantes Prata, Louis Nakayama Ohe, Kelvin Henrique Vilalva, Lucas Ferreira Marcondes Lemos, Paola Emanuela Poggio Smanio

**Affiliations:** 1 Instituto Dante Pazzanese de Cardiologia São Paulo SP Brasil Instituto Dante Pazzanese de Cardiologia, São Paulo, SP – Brasil; 2 Fleury Group São Paulo SP Brasil Fleury Group, São Paulo, SP – Brasil

**Keywords:** Angina Instável, Dor no Peito, Síndrome Coronariana Aguda

## Abstract

**Fundamento:**

O manejo da angina instável (AI) é um desafio devido ao seu diagnóstico subjetivo e à sua escassa representação em ensaios clínicos randomizados que determinem as práticas atuais.

**Objetivos:**

O objetivo deste estudo é identificar os principais fatores associados à indicação de estratificação invasiva ou não nessa população e avaliar os fatores associados às alterações nos exames de estratificação.

**Métodos:**

Coorte retrospectiva de pacientes internados por AI, em um período de 20 meses consecutivos. Para avaliar os fatores associados à estratégia de estratificação, os pacientes foram divididos em estratificação invasiva (cinecoronariografia) e não invasiva (demais métodos). Para análise de fatores relacionados às alterações nos exames de estratificação, os pacientes foram divididos em grupos com ou sem doença arterial coronariana (DAC) obstrutiva ou isquemia, conforme resultados dos exames solicitados. Foram realizadas comparações entre grupos e análise de regressão logística múltipla, com significância estatística definida em um nível de 5%.

**Resultados:**

729 pacientes foram incluídos, com mediana de idade de 63 anos e predomínio do sexo masculino (64,6%). Estiveram associados à estratificação invasiva: tabagismo (p = 0,001); tipo de dor torácica (p < 0,001); dor "em crescendo" (p = 0,006); escore TIMI (p = 0,006); escore HEART (p = 0,011). Na análise multivariada, tabagistas (OR 2,23, IC 95% 1,13-4,8), ex-tabagistas (OR 2,19, IC 1,39-3,53) e dor torácica tipo A (OR 3,39, IC 95% 1,93-6,66) estiveram associados de forma independente. Estiveram associados à DAC obstrutiva ou isquemia: tempo de internação hospitalar (p < 0,001); sexo masculino (p = 0,032); dor desencadeada por esforço (p = 0,037); Diamond-Forrester (p = 0,026); escore TIMI (p = 0,001). Na análise multivariada, apenas dor torácica (dor torácica tipo B: OR 0,6, IC 95% 0,38-0,93, p = 0,026) e DAC prévia (OR 1,42, IC 95% 1,01-2,0, p = 0,048) estiveram associadas de maneira independente.

**Conclusões:**

O tipo de dor torácica desempenha um papel crucial não apenas no diagnóstico da AI, mas também na definição do tratamento adequado. Nossos resultados destacam a importância de incorporar características da dor aos escores prognósticos endossados pelas diretrizes, para otimização do manejo da AI.

**Figure:**
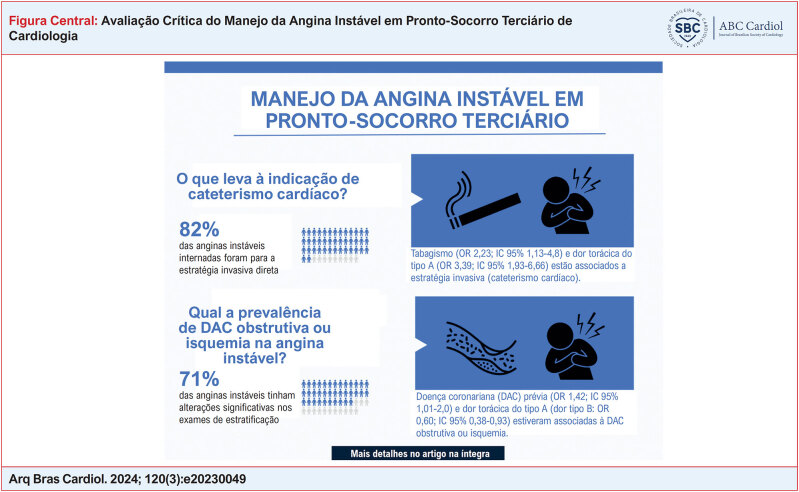


## Introdução

A doença cardiovascular é a principal causa de morbimortalidade no Brasil e no mundo, representando cerca de 30% de todos os óbitos, conforme dados do *Global Burden Disease* de 2019.^1^ A doença coronariana no Brasil, nesse mesmo ano, foi responsável por cerca de 288.000 internações na rede pública, com um total de 16.880 óbitos, de acordo com o *DATASUS* .^2^

Pela ausência de um marcador objetivo para definir o quadro de angina instável (tais quais os marcadores de necrose para definir infarto agudo), o diagnóstico dessa entidade é eminentemente clínico, com auxílio do eletrocardiograma.^3^ Assim, abre espaço para a subjetividade e dificuldade na tomada de conduta pelos serviços de saúde.

A documentação de injúria miocárdica com troponina positiva leva a um caminho já bem estabelecido: cateterismo cardíaco precoce e intervenção coronária percutânea se necessário. A ausência desse marcador, portanto, já não demonstra clara evidência dessa intervenção em nenhum cenário dos grandes ensaios clínicos randomizados (ECR).^4 - 7^

Saber identificar qual angina instável se beneficia de tratamento invasivo é uma das questões ainda não respondidas na literatura. Assim, se torna de fundamental importância a continuidade do estudo sobre o assunto, para tentar entender os motivos que levam à indicação da cinecoronarionagrafia e se ainda é possível encontrarmos algum outro marcador que possa sugerir necessidade de intervenção coronariana.

Com o desenvolvimento de biomarcadores cada vez mais sensíveis para o diagnóstico do Infarto Agudo do Miocárdio, a incidência de Angina Instável vem diminuindo consideravelmente. A diretriz europeia mais recente praticamente desconsidera o termo, sugerindo a estratificação de uma síndrome coronariana aguda (SCA) com troponina ultrassensível negativa através de métodos não invasivos.^8^ Apesar da tendência a esquecer o termo angina instável,^3^ os pronto-atendimentos permanecerão recebendo pacientes com quadro clínico sugestivo de SCA, porém com troponina negativa, principalmente em centros que não dispõem da troponina ultrassensível.

O estudo do manejo da angina instável em um pronto-socorro terciário de cardiologia, com laboratório de hemodinâmica disponível e de fácil acesso; e a pesquisa de fatores relacionados à necessidade de intervenção na Angina Instável; não somente podem contribuir para o entendimento dessa doença, como também na redução de procedimentos desnecessários, otimização de fluxos nas instituições e manejo de recursos na realidade brasileira. O objetivo primário do estudo é avaliar o manejo da angina instável em um pronto-socorro terciário de cardiologia e o secundário avaliar fatores associados à presença de doença arterial coronariana (DAC) obstrutiva ou isquemia pelos resultados dos exames realizados na estratificação.

## Métodos

O presente estudo trata-se de uma coorte retrospectiva com o intuito de identificar fatores associados à indicação da estratégia invasiva na angina instável, elaborada através da análise do banco de dados de um pronto-socorro terciário de cardiologia. Os dados foram coletados através da coorte "Registro dos casos de síndrome coronariana aguda no Pronto-Socorro".

Dados adicionais foram extraídos do sistema de atendimento eletrônico de um pronto-socorro terciário de cardiologia, com avaliação complementar de prontuários físicos para laudos dos exames solicitados.

Foram analisados os dados do período de 16 de julho de 2018 a 28 de fevereiro de 2020.

A pesquisa foi aprovada pelo Comitê de Ética e Pesquisa da instituição, com o parecer número 4.711.692, de 14 de maio de 2021, estando de acordo com as atribuições definidas na resolução CNS nº 466 de dezembro de 2012, sobre Diretrizes e Normas Regulamentadoras de Pesquisa Envolvendo Seres Humanos, do Conselho Nacional de Saúde / Agência Nacional de Vigilância Sanitária e as Boas Práticas de Pesquisa Clínica do ICH-GCP.

### Critérios de Inclusão e Exclusão

Foram incluídos no estudo todos os pacientes internados com diagnóstico final de Angina Instável no período estabelecido, de maneira consecutiva.

Foram excluídos pacientes que não tinham história clínica condizente com angina instável, definida como: angina de início recente (classe II ou III pela classificação da Canadian Cardiovascular Society, com início nos últimos 2 meses), angina em crescendo (piora progressiva de intensidade e/ou frequência) e angina em repouso.^9 - 13^ Foram excluídos pacientes com história sugestiva de angina pós infarto.

Foram excluídos pacientes com curva de troponina positiva. A troponina utilizada no período do estudo era a Troponina T convencional (c-TnT).

Foram excluídos do estudo pacientes que não realizaram nenhuma estratificação da SCA na internação.

Foram excluídos pacientes que, após análise do eletrocardiograma, apresentavam alterações sugestivas de supradesnivelamento do segmento ST.

### Desenho

Para a análise do objetivo primário, os pacientes foram divididos conforme o método de estratificação inicial, formando grupos de estratificação invasiva (cinecoronariografia) e não invasiva (cintilografia perfusional do miocárdio ou angiotomografia de coronárias). Os dados clínicos-laboratoriais, epidemiológicos, fatores de risco, escores prognósticos e calculadoras pré-teste para DAC foram avaliados individualmente quanto à presença ou não de associação através de análises de regressão. Os métodos de estratificação realizados (cinecoronariografia, cintilografia de perfusão do miocárdio com estresse ou angiotomografia de coronárias) foram realizados conforme protocolo institucional específico de cada setor.

Como análise secundária, os pacientes foram divididos entre presença ou não de "DAC Obstrutiva ou Isquemia". "DAC Obstrutiva ou Isquemia" foi definida conforme resultado dos exames de estratificação solicitados:

1. Cinecoronariografia com estenose maior ou igual a 70% ou necessidade de implante de *stent* (conforme julgamento da equipe assistente);
2. Angiotomografia de Coronárias com estenose significativa do lúmen coronariano conforme laudo institucional (> 50% de obstrução);
3. Cintilografia do Miocárdio com presença de hipocaptação transitória sugestiva de isquemia (carga isquêmica) ou com achados de alto risco (queda na fração de ejeção > 10%; dilatação transitória do ventrículo esquerdo e/ou captação pulmonar ou de ventrículo direito).

### Variáveis analisadas

Foram avaliados os seguintes fatores relacionados às características de base dos pacientes: idade; sexo (masculino ou feminino); índice de massa corpórea; hipertensão arterial sistêmica; diabetes mellitus (insulinodependente ou não); dislipidemia; tabagismo ou ex-tabagista; taxa de filtração glomerular e presença ou não de doença renal crônica (clearence de creatinina < 60 ml/min); DAC prévia (por meio de descrição em prontuário eletrônico).

Quanto ao eletrocardiograma, foi discriminado, conforme laudo institucional, em: eletrocardiograma normal, presença de alterações difusas de repolarização, presença de alterações sugestivas de isquemia (alterações de onda T), presença de área eletricamente inativa e infradesnivelamento de ST. Qualquer outra alteração no eletrocardiograma (sobrecargas, bloqueios de ramo...) foram discriminados na categoria "outros".

Escores prognósticos (TIMI, GRACE e HEART) e calculadora de probabilidade pré-teste de DAC (Diamond Forrrester), também foram estudados como variáveis contínuas.

#### Características da dor torácica na emergência

Para diferenciar o tipo de dor torácica na emergência, foram coletados os seguintes dados:

- Tipo de dor, conforme descrita pelo estudo CASS:^14^ 1) tipo A - definitivamente anginosa (tem todas as características de angina como aperto retroesternal, irradiação para membros superiores e/ou pescoço, piora ou desencadeada por esforço, alívio com repouso e/ou nitrato); 2) tipo B – provavelmente anginosa (tem a maioria, mas não todas as características anginosas); 3) tipo C – provavelmente não anginosa (tem uma ou outra característica anginosa, com manifestações atípicas);
- Dor desencadeada ao esforço ou com início em repouso;
- Presença de sintomas associados: sudorese e/ou náuseas / vômitos;
- Relato de a dor ser semelhante ao quadro de SCA prévia;
- Dor em piora progressiva de frequência, intensidade ou desencadeada por esforços cada vez menores (em crescimento).

### Análise estatística

As variáveis contínuas foram descritas através de mediana e intervalo interquartil e as variáveis categóricas foram apresentadas através de frequência e porcentagem. A comparação entre os grupos foi realizada através do teste Qui-Quadrado (variáveis categóricas) ou do teste de Mann-Whitney (variáveis contínuas). Os fatores associados aos desfechos foram avaliados através de Regressão Logística Múltipla com critério de seleção de variáveis *stepwise* . O nível de significância considerado nas análises foi de 5%. O software utilizado foi "The R Foundation for Statistical Computing, versão 4.2.0. 2022". O teste de Shapiro-Wilk foi usado para testar a normalidade das variáveis contínuas, nenhuma apresentou variável normal.

## Resultados

De 16 de julho de 2018 a 28 de fevereiro de 2020, 898 pacientes foram internados com diagnóstico final de angina instável pelo CID-10 da internação. Após revisão de história clínica, eletrocardiograma e outros critérios de exclusão, obteve-se uma amostra de 729 pacientes. Houve predomínio do sexo masculino (64,6%), com idades entre 33 e 91 anos, média de 62,9. A prevalência das principais comorbidades foram: 82% de hipertensos, 46% de diabéticos, 58% de dislipidêmicos, 18% tabagistas, 19% com doença renal crônica e 62% com doença coronariana prévia.

### Avaliação do método de estratificação

A Tabela 1 apresenta a comparação entre os grupos de estratificação invasiva e não invasiva, considerando todas as variáveis analisadas. Dos 729 participantes, 81,7% foram estratificados de maneira invasiva (cateterismo cardíaco como primeiro exame). Dos 133 pacientes estratificados de maneira não invasiva, 96 (72,2%) realizaram cintilografia de perfusão do miocárdio, 31 (23,3%) realizaram angiotomografia de coronárias, 2 (1,5%) realizaram ressonância magnética de coração com estresse e 4 (3%) realizaram teste ergométrico.

**Tabela 1:** Características de base e associação com método de estratificação

Variável			Método de Estratificação		p-valor
			Invasiva (N=596)	Não invasiva (N=133)	
**Antecedentes**					
	Idade, anos (mediana, [Q1;Q3])		63 [56;69]	63 [55;71]	0,966
	Sexo masculino		64,4%	65,4%	0,909
	IMC>30		28,4%	30,8%	0,643
	HAS		82,4%	81,2%	0,845
	DM		45,6%	50,4%	0,371
	DM ID		10,9%	15,0%	0,357
	DLP		59,4%	51,9%	0,136
	DRC		18,8%	18,8%	1,000
	Tabagismo ativo		13,6%	8,27%	0,001
	DAC Prévia		62,2%	60,2%	0,725
**Dor Torácica**					
	Classificação				<0,001
		A	25,1%	11,5%	
		B	48,5%	48,1%	
		C	26,4%	40,5%	
	Repouso / Esforço				0,127
		Esforço	34,0%	26,6%	
		Repouso	66,0%	73,4%	
	Em crescendo		26,8%	15%	0,006
**Escores**					
	GRACE (mediana, [Q1;Q3])		92,5 [75;110]	92 (24)	0,881
	TIMI (mediana, [Q1;Q3])		4 [3;4]	3 [2;4]	0,006
	HEART (mediana, [Q1;Q3])		5 [5;6]	5 [4;6]	0,011
**ECG normal**			108 (18,1%)	28 (21,1%)	0,508
**ADRV**			166 (27,9%)	36 (27,1%)	0,940
**AEI**			93 (15,6%)	18 (13,5%)	0,640
**Isquemia**			72 (12,1%)	9 (6,77%)	0,107
**Infra ST (mm)**					0,660
	0		584 (98,0%)	133 (100%)	
	0.5		1 (0,17%)	0 (0,00%)	
	1		9 (1,51%)	0 (0,00%)	
	2		2 (0,34%)	0 (0,00%)	

Fonte: elaborado pelo próprio autor.

IMC: índice de massa corpórea; HAS: hipertensão arterial sistêmica; DM: diabetes mellitus; DM ID: diabetes mellitus insulinodependente; DLP: dislipidemia; DRC: doença renal crônica; DAC: doença arterial coronariana; ECG: eletrocardiograma; ADRV: alterações difusas da repolarização ventricular; AEI: área eletricamente inativa; Infra ST: infradesnivelamento do segmento ST.

O grupo de estratificação invasiva apresenta mais tabagistas e ex-tabagistas (p-valor = 0,001) do que o grupo de estratificação não invasiva. Não houve diferença estatística nas demais características de base associadas, incluindo comorbidades como diabetes, hipertensão ou presença de DAC prévia conhecida.

Quanto às características da dor torácica, as únicas variáveis associadas à maior chance de cateterismo cardíaco foram: dor do tipo A (p-valor < 0,001) e dor com característica "em crescendo" (p-valor = 0,006). Nenhuma das outras variáveis relacionadas à dor foram estatisticamente significativas para a associação.

Não houve diferença estatística em nenhuma variável eletrocardiográfica estudada. Alterações sugestivas de isquemia foram encontradas em 12,1% dos pacientes que foram submetidos ao cateterismo, em contraste a 6,77% dos pacientes com estratificação conservadora (p-valor = 0,107). Nenhum paciente com infradesnivelamento do segmento ST no eletrocardiograma de admissão foi para estratificação não invasiva.

As associações dos escores de risco com a indicação de estratificação invasiva também são demonstradas ao fim da Tabela 1 . Tanto o escore TIMI (p-valor = 0,006) quanto o escore HEART (p-valor = 0,011) tiveram associação com a estratificação invasiva. O HEART, porém, teve o mesmo valor de mediana. A mediana do escore GRACE foi de 92.5 no grupo de estratificação invasiva e 91.0 no de não invasiva. Apenas 3,3% (24 pacientes) de toda amostra apresentava escore GRACE maior do que 140, sendo 92% (22) deles estratificados com cateterismo cardíaco. 26% dos pacientes apresentavam o escore GRACE entre 109 e 140, com 71% sendo estratificados de maneira invasiva.

Para avaliar os fatores que, conjuntamente, estão mais associados ao método de estratificação, todas as variáveis das tabelas acima foram colocadas em um modelo de regressão logística múltipla, demonstrado na Tabela 2 . As variáveis que melhor explicam a chance de cateterismo cardíaco como estratégia inicial são tabagismo e o tipo de dor. Pacientes tabagistas ativos e ex-tabagistas tiveram 2,23 e 2,19 vezes mais chances, respectivamente, de serem estratificados com estratégia invasiva do que pacientes que nunca fumaram. Pacientes com dor torácica tipo A tiveram 3,39 vezes mais chances de cateterismo como estratificação inicial do que pacientes com dor tipo C.

**Tabela 2:** Variáveis dos pacientes que isoladamente apresentam maior chance de estratificação invasiva

Variável	Categoria	OR	IC95%	p-valor
Tabagismo	Ativo	2,23	1,13-4,80	0,028
	Parou < 6m	0,84	0,41-1,85	0,646
	Ex	2,19	1,39-3,53	0,001
Tipo de Dor	A	3,39	1,83-6,66	<0,001
	B	1,48	0,97-2,27	0,070

Fonte: elaborado pelo próprio autor.

Ao considerar as indicações da diretriz brasileira de 2021^9^ para a estratificação invasiva (Tabela 1, capítulo 1.4), 94,3% de toda a amostra deveria ir para a estratificação inicial invasiva. Ao considerar as indicações da diretriz europeia de 2020,^8^ 15,5% da amostra deveria ir para o cateterismo cardíaco como estratégia inicial.

### Avaliação de "DAC Obstrutiva ou Isquemia" na população

Após análises dos resultados dos exames de estratificação inicial realizados nos 729 participantes, 520 (71,3%) apresentavam alterações sugestivas de "DAC Obstrutiva ou Isquemia". A Tabela 3 demonstra a comparação entre os grupos com e sem "DAC Obstrutiva ou Isquemia", considerando todas as variáveis analisadas. Pacientes com "DAC Obstrutiva ou Isquemia" tiveram uma mediana de dois dias a mais de internação hospitalar (p-valor < 0.001). O grupo com "DAC Obstrutiva ou Isquemia", na análise univariada, apresentou maior prevalência de pacientes do sexo masculino (p-valor = 0,032). Nenhuma das demais variáveis relacionadas às características de base tiveram diferença estatística. A presença de DAC prévia conhecida esteve um pouco mais prevalente no grupo com "DAC Obstrutiva ou Isquemia", porém sem significância (63,5% *versus* 57,9%; p-valor = 0,188). Observa-se maior prevalência de DRC no grupo com os exames alterados, que também não atingiu a significância estatística (20,4% *versus* 14,8%; p-valor = 0,103).

**Tabela 3:** Características de base e associação com "DAC Obstrutiva ou Isquemia" nos exames de estratificação

Variável			DAC Obstrutiva ou Isquemia		p-valor
			Não (N = 209)	Sim (N = 520)	
**Antecedentes**					
	Idade, anos(mediana, [Q1;Q3])		63 [55,0;70,0]	63 [56,0;70,0]	0.399
	Sexo masculino		58,4%	67,1%	0,032
	IMC > 30 kg/m^2^		30,1%	28,3%	0,678
	HAS		84,7%	81,2%	0,307
	DM		48,3%	45,8%	0,587
	DM insulinodependente		9,57%	12,5%	0,277
	DLP		60,8%	56,9%	0,386
	DRC		14,8%	20,4%	0,103
	Tabagismo ativo		11%	13,3%	0,439
	DAC prévia		57,9%	63,5%	0,188
**Dor torácica**					
	Classificação				0,105
		A	17,4%	24,7%	
		B	51,7%	47,1%	
		C	30,9%	28,2%	
	Repouso / Esforço				0,037
		Exercício	26,7%	35,1%	
		Repouso	73,3%	64,9%	
	Em crescendo		20,6%	26,3%	0,124
**Escores**					
	GRACE(mediana, [Q1;Q3])		91 [74;111]	92 [75;109]	0,494
	TIMI(mediana, [Q1;Q3])		3 [2;4]	4 [3;4]	0,001
	HEART (mediana, [Q1;Q3])		5 [4;6]	5 [4;6]	0,542
**ECG normal**			16,7%	19,4%	0,463
**ADRV**			30,6%	26,5%	0,307
**AEI**			11,5%	16,7%	0,095
**Isquemia**			8,13%	12,3%	0,136
**Infra ST**					0,646
	0		99,5%	97,9%	
	0,5		0,00%	0,19%	
	1		0,48%	1,54%	
	2		0,00%	0,38%	

Fonte: elaborado pelo próprio autor.

IMC: índice de massa corpórea; HAS: hipertensão arterial sistêmica; DM: Diabetes mellitus; DM ID: Diabetes mellitus insulinodependente; DLP: Dislipidemia; DRC: doença renal crônica; DAC: doença arterial coronariana; ECG: eletrocardiograma; ADRV: alterações difusas da repolarização ventricular; AEI: área eletricamente inativa; Infra ST: infradesnivelamento do segmento ST.

A única variável relacionada à característica da dor torácica que, na análise univariada, esteve associada significativamente com "DAC Obstrutiva ou Isquemia" foi a dor desencadeada por esforço (p-valor = 0,037). Dor torácica tipo A (24,7% *versus* 17,4%; p-valor = 0,105) e característica "em crescendo" da dor (26,3% *versus* 20,6%; p-valor = 0,124), estiveram mais prevalentes no grupo "DAC Obstrutiva ou Isquemia", sem atingir significância estatística.

Não houve diferença estatística em nenhuma variável eletrocardiográfica estudada. Pacientes com os resultados positivos nos exames de estratificação apresentaram tendência a maior prevalência de isquemia e área eletricamente inativa no ECG, mas sem conseguir atingir significância estatística.

Quanto aos escores prognósticos, nota-se que o grupo com "DAC Obstrutiva ou Isquemia" apresentou maior valor no escore TIMI (mediana 4 *versus* 3; p-valor = 0,001), único escore prognóstico que apresentou diferença estatística.

Para avaliar os fatores que, conjuntamente, estiveram mais associados à "DAC Obstrutiva ou Isquemia", todas as variáveis da Tabela 1 foram colocadas em um modelo de regressão logística múltipla. Como mostra a Tabela 4 , as variáveis que, após análise multivariada, melhor explicam a chance de alteração nos exames de estratificação iniciais sugestivas de "DAC Obstrutiva ou Isquemia" são: classificação da dor torácica e a presença de DAC prévia.

**Tabela 4:** Variáveis dos pacientes que isoladamente apresentam maior chance de "DAC Obstrutiva ou Isquemia" nos exames de estratificação

Variável	Categoria	OR	IC95%	p-valor
Classificação	B	0,60	0,38-0,93	0,026
	C	0,61	0,37-1,00	0,051
DAC prévia	Sim	1,42	1,01-2,00	0,048
TIH		1,13	1,08-1,19	<0,001

Fonte: elaborado pelo próprio autor.

DAC: doença arterial coronariana. TIH: tempo de internação hospitalar.

Pacientes com dor torácica classificada como tipo A tem chance 66% (1/0,6) maior de DAC que pacientes classificados como tipo B; pacientes com DAC prévia tem chance 42% maior de DAC que pacientes sem DAC prévia. A presença de "DAC Obstrutiva ou Isquemia" também esteve associada à maior a maior tempo de internação hospitalar na análise multivariada.

## Discussão

O primeiro ponto de destaque para os dados em evidência é respeito ao perfil de pacientes internados com angina instável no serviço estudado. Percebemos uma prevalência aumentada de comorbidades classicamente associadas à DAC. Enquanto o estudo *FRISC II* ,^6^ um dos principais ECR de estratificação de SCASSST, apresentava uma prevalência de 30% de hipertensos, 13% de diabéticos e 23% com infarto prévio, a população estudada nesse centro terciário apresentava 80% de hipertensos, 47% de diabéticos e 60% com doença coronariana prévia. Certamente não se trata de um perfil da população brasileira, mas sim da complexidade dos pacientes de um pronto-socorro aberto de serviço terciário do sistema público de saúde.

Ainda com relação às características de base, era plausível a expectativa de que uma maior prevalência dessas comorbidades auxiliasse na indicação do cateterismo no cenário da emergência, visto tanto pelo aumento da probabilidade de DAC quanto pelas orientações das diretrizes vigentes na época.^10 , 11^ Porém a única comorbidade mais prevalente no grupo da estratificação invasiva foi o tabagismo. Ou seja, o paciente com algum histórico de tabagismo era tendenciado à estratégia invasiva direta na admissão do pronto-socorro.

Ao analisarmos a prevalência das comorbidades com a presença de DAC obstrutiva ou isquemia nos exames, a amostra estudada apresenta uma peculiaridade: apenas o sexo masculino esteve em associação independente. Na análise multivariada, a presença de DAC prévia, como esperado e com boa plausibilidade, esteve em associação com a DAC obstrutiva na angina instável. O fato de HAS, DM, TBG e outras comorbidades não estarem associados à DAC obstrutiva pode ser um viés da população estudada pela alta prevalência dessas comorbidades como comentado anteriormente. Ou seja, a tendência de indicar o cateterismo para tabagistas não foi refletida em alterações nos exames.

As alterações eletrocardiográficas sugestivas de isquemia no ECG da admissão foram poucas, não conseguindo atingir significância estatística em nenhum dado estudado. Porém os números relativos mostram uma tendência à maior prevalência de área eletricamente inativa e de alterações isquêmicas em pacientes com DAC obstrutiva, o que pode reforçar as orientações das diretrizes em indicar cateterismo cardíaco nesses pacientes. O eletrocardiograma tem um grande valor prognóstico, principalmente na presença de infradesnivelamento do segmento ST, que inclusive é um dos critérios do escore TIMI,^12^ além de ter sido identificado como critério de alto risco no estudo *TATICS-TIMI 18* .^5^ As diretrizes^8 , 9^ reforçam a indicação da estratégia invasiva direta no caso da presença dessa alteração eletrocardiográfica e foi observado na amostra estudada que 100% dos pacientes com infradesnivelamento do segmento ST tiveram o cateterismo cardíaco como estratificação inicial.

Quanto aos escores diagnósticos, estiveram associados estatisticamente o escore TIMI e o escore HEART para indicação da estratificação invasiva como estratégia inicial. Porém a relevância clínica dessa associação parece ser pouco significante uma vez que a diferença das medianas é muito pequena (3 para 4 no TIMI e 5 para 5 no HEART, apesar da diferença no p-valor). E a diferença identificada no HEART está provavelmente relacionada ao tipo de dor, variável que é incluída nessa calculadora, uma vez que na análise multivariada, ambos os escores não se mostram indicativos. O mesmo raciocínio serve para associação encontrada do TIMI com a DAC obstrutiva ou isquemia: apesar de significância estatística, a significância clínica da diferença encontrada é mínima.

Chamam muito a atenção os resultados referentes ao escore GRACE. Pela diretriz europeia atual,^8^ deveríamos indicar cateterismo cardíaco na angina instável como estratégia inicial apenas para pacientes com escore GRACE maior que 140, alterações eletrocardiográficas ou com algum grau de instabilidade. E apenas 3,3% da população geral de angina instável era de alto risco por esse escore, demonstrando a grande disparidade entre o que é dito nas diretrizes e o que é realizado na prática clínica. Além disso, apesar de ter se demonstrado um excelente preditor de eventos nos estudos em que foi validado,^13^ ele não esteve associado à presença de DAC obstrutiva ou isquemia nos resultados dos exames complementares.

Apesar das diretrizes, tanto a brasileira como a europeia, sugerirem um fluxo para manejo da angina instável, percebemos uma diferença importante entre a orientação das diretrizes e o que ocorre em um centro de referência (terciário) do serviço público de saúde brasileiro. Enquanto pela diretriz brasileira de 2021 94% da amostra deveria ter sido estratificada por cateterismo (em sua maioria pela indicação de cateterismo em pacientes de risco intermediário),^9^ pela diretriz europeia de 2020,^8^ apenas 15%. E nenhuma delas cita a característica da dor torácica como critério para escolha do método de estratificação. Além disso, na amostra do presente estudo, foi o tipo de dor a variável mais relevante na escolha da estratégia: pacientes com dor definitivamente anginosa tiveram 3,39 vezes mais chances de ir para o cateterismo cardíaco e não o uso de escores prognósticos.

Surpreendentemente, o achado da presença de DAC prévia não influenciou na escolha do método de estratificação nesses pacientes internados com AI. Uma possível explicação para o fato se deve à alta prevalência de DAC nessa população (62%), o que pode enviesar os achados.

Ao analisarmos a prevalência da DAC obstrutiva ou isquemia em homens com dor do tipo A (79%), percebemos uma aproximação com os dados do estudo *CASS* ,^14^ que em 1989 descreveu uma prevalência de 93% de DAC obstrutiva nos homens com esse tipo de dor. O estudo *CASS* excluiu síndromes coronarianas agudas, mas a semelhança entre os achados reforça o conceito do "continuum" cardiovascular, de que a doença coronariana é uma só e que as diferenças entre seus estágios são mais prognósticas do que fisiopatológicas.

Uma dor torácica tipo A (com todas as características sugestivas de SCA) esteve associado a uma chance de 66% a mais de estarmos diante de uma DAC obstrutiva ou com isquemia. Enquanto as diretrizes recomendam a estratificação invasiva apenas em pacientes classificados como risco intermediário^9^ ou alto,^8^ guiar o tipo de estratificação pelo tipo de dor pode otimizar o manejo da angina instável.

Uma das principais limitações do estudo é o viés da amostra estudada, visto se tratar de um estudo unicêntrico e terciário, de complexidade aumentada. Não devemos extrapolar os achados para outras populações. E, pelo tipo de desfecho analisado, não se podem extrapolar os achados para a visão de benefício clínico, uma vez que o desfecho analisado foi a alteração no exame de estratificação e não desfechos duros como mortalidade ou eventos cardiovasculares. A resposta na literatura sobre o real benefício clínico do cateterismo cardíaco direto na angina instável (troponina negativa) ainda permanece incerta.

## Conclusão

Os fatores associados de maneira independente à indicação da estratificação invasiva de angina instável em um centro terciário de cardiologia no sistema público de saúde brasileiro foram tabagismo e classificação da dor torácica. Os fatores associados de maneira independente à DAC obstrutiva ou isquemia foram presença de DAC prévia e classificação da dor torácica.

Nossos resultados enfatizam a importância da avaliação da dor torácica não somente para o diagnóstico da angina instável, mas também no seu uso para definição do método de estratificação.
