# Supplementary material for: Avaliação Crítica do Manejo da Angina Instável em Pronto-Socorro Terciário de Cardiologia
Source: Arq Bras Cardiol. 2024 Mar 21;121(3):e20230049. [Article in Portuguese] doi: 10.36660/abc.20230049 (PMC12092038; doi:10.36660/abc.20230049)
Supplement: Translation

# Translation: Critical Assessment of the Management of Unstable Angina in a Specialized Cardiology Emergency Room

**DOI:** 10.36660/abc.20230049i

**Authors:** Mateus Arantes Prata, Louis Nakayama Ohe, Kelvin Henrique Vilalva, Lucas Ferreira Marcondes Lemos, Paola Emanuela Poggio Smanio

**Affiliations:** 1 São Paulo SP Brazil Instituto Dante Pazzanese de Cardiologia, São Paulo, SP – Brazil; 2 São Paulo SP Brazil Fleury Group, São Paulo, SP – Brazil

**Keywords:** Unstable Angina, Chest Pain, Acute Coronary Syndrome

## Abstract

**Background:**

The management of unstable angina (UA) presents a challenge due to its subjective diagnosis and limited representation in randomized clinical trials that inform current practices.

**Objectives:**

This study aims to identify key factors associated with the indication for invasive versus non-invasive stratification in this population and to evaluate factors associated with stratification test results.

**Methods:**

This retrospective cohort study included patients hospitalized with UA over a consecutive 20-month period. To assess factors associated with stratification strategies, patients were divided into invasive stratification (coronary angiography) and non-invasive stratification (other methods) groups. For the analysis of factors related to changes in stratification tests, patients were categorized into groups with or without obstructive coronary artery disease (CAD) or ischemia, as per the results of the requested tests. Comparisons between groups and multiple logistic regression analyses were performed, with statistical significance set at a 5% level.

**Results:**

A total of 729 patients were included, with a median age of 63 years and a predominance of males (64.6%). Factors associated with invasive stratification included smoking (p = 0.001); type of chest pain (p < 0.001); "crescendo" pain (p = 0.006); TIMI score (p = 0.006); HEART score (p = 0.011). In multivariate analysis, current smokers (OR 2.23, 95% CI 1.13-4.8), former smokers (OR 2.19, 95% CI 1.39-3.53), and type A chest pain (OR 3.39, 95% CI 1.93-6.66) were independently associated. Factors associated with obstructive CAD or ischemia included length of hospital stay (p < 0.001); male gender (p = 0.032); effort-induced pain (p = 0.037); Diamond-Forrester score (p = 0.026); TIMI score (p = 0.001). In multivariate analysis, only chest pain (type B chest pain: OR 0.6, 95% CI 0.38-0.93, p = 0.026) and previous CAD (OR 1.42, 95% CI 1.01-2.0, p = 0.048) were independently associated.

**Conclusion:**

The type of chest pain plays a crucial role not only in the diagnosis of UA but also in determining the appropriate treatment. Our results highlight the importance of incorporating pain characteristics into prognostic scores endorsed by guidelines to optimize UA management.

## Introduction

Cardiovascular disease is the leading cause of morbidity and mortality in Brazil and worldwide, accounting for about 30% of all deaths, according to the 2019 Global Burden of Disease data.^1^In Brazil, coronary heart disease alone was responsible for approximately 288,000 hospital admissions in the public network in the same year, with a total of 16,880 deaths, as reported by DATASUS.^2^

Due to the absence of an objective marker to define unstable angina (such as necrosis markers for acute myocardial infarction), the diagnosis of this entity is predominantly clinical, aided by electrocardiogram.^3^This opens up room for subjectivity and difficulty in decision-making by healthcare services.

Documentation of myocardial injury with positive troponin leads to a well-established path: early cardiac catheterization and percutaneous coronary intervention if necessary. The absence of this marker, however, does not demonstrate clear evidence of this intervention in any scenario of the major randomized clinical trials (RCTs).^4 - 7^

Identifying which unstable angina benefits from invasive treatment is one of the questions still unanswered in the literature. Therefore, continuing to study this subject is of fundamental importance to try to understand the reasons leading to the indication of coronary angiography and whether it is still possible to find some other marker that may suggest the need for coronary intervention.

With the development of increasingly sensitive biomarkers for the diagnosis of Acute Myocardial Infarction, the incidence of Unstable Angina has been decreasing significantly. The most recent European guideline virtually disregards the term, suggesting stratification of an acute coronary syndrome (ACS) with negative high-sensitivity troponin through non-invasive methods.^8^Despite the trend to overlook the term unstable angina,^3^emergency departments will continue to receive patients with clinical symptoms suggestive of ACS but with negative troponin, especially in centers that do not have high-sensitivity troponin available.

The study of the management of unstable angina in a tertiary cardiology emergency department, with an available and easily accessible hemodynamics laboratory, and the research of factors related to the need for intervention in Unstable Angina cannot only contribute to the understanding of this disease but also in reducing unnecessary procedures, optimizing flows in institutions, and managing resources in the Brazilian reality. The primary objective of the study is to assess the management of unstable angina in a tertiary cardiology emergency department, and the secondary is to evaluate factors associated with the presence of obstructive coronary artery disease (CAD) or ischemia by the results of the tests performed in the stratification.

## Methods

This study is a retrospective cohort aimed at identifying factors associated with the indication of an invasive strategy in unstable angina, developed through the analysis of a database from a tertiary cardiology emergency department. The data were collected through the cohort "Registry of Acute Coronary Syndrome Cases in the Emergency Department".

Additional data were extracted from the electronic service system of a tertiary cardiology emergency department, with further evaluation of physical medical records for reports of the requested examinations.

Data from the period of July 16, 2018, to February 28, 2020, were analyzed.

The Ethics and Research Committee of the institution approved the research under opinion number 4.711.692, dated May 14, 2021. It complies with the attributions defined in the CNS Resolution No. 466 of December 2012 on Guidelines and Regulatory Standards for Research Involving Human Beings from the National Health Council/National Health Surveillance Agency and the Good Clinical Research Practices of ICH-GCP.

### Inclusion and exclusion criteria

The study included all patients admitted with a final diagnosis of Unstable Angina during the established period consecutively.

Patients who did not have a clinical history consistent with unstable angina, defined as newly onset angina (class II or III by the Canadian Cardiovascular Society classification, starting in the last 2 months), crescendo angina (progressive worsening of intensity and/or frequency), and rest angina,^9 - 13^were excluded. Patients with a history suggestive of post-infarction angina were also excluded.

Patients with a positive troponin curve were excluded. The troponin used during the study period was conventional Troponin T (c-TnT).

Patients who did not undergo any stratification of ACS during admission were excluded from the study.

Patients who, after analysis of the electrocardiogram, showed changes suggestive of ST-segment elevation were excluded.

### Design

For the analysis of the primary objective, patients were divided according to the initial stratification method, forming groups of invasive stratification (coronary angiography) and non-invasive stratification (myocardial perfusion scintigraphy or coronary angiography). Clinical-laboratory, epidemiological data, risk factors, prognostic scores, and pre-test calculators for CAD were individually assessed for the presence or absence of association through regression analyses. The stratification methods performed (coronary angiography, myocardial perfusion scintigraphy with stress, or coronary angiography) were carried out according to the specific institutional protocol of each department.

As a secondary analysis, patients were divided between the presence or absence of "Obstructive CAD or Ischemia". "Obstructive CAD or Ischemia" was defined according to the results of the requested stratification exams:

1. Coronary angiography with stenosis greater than or equal to 70% or need for stent implantation (as judged by the attending team);
2. Coronary Angiotomography with significant narrowing of the coronary lumen as per institutional report (> 50% obstruction);
3. Myocardial Scintigraphy with the presence of transient hypo-uptake suggestive of ischemia (ischemic burden) or with high-risk findings (drop in ejection fraction > 10%; transient dilation of the left ventricle and/or pulmonary or right ventricle uptake).

### Variables

The following factors related to the baseline characteristics of the patients were evaluated: age; gender (male or female); body mass index; systemic arterial hypertension; diabetes mellitus (insulin-dependent or not); dyslipidemia; smoking or former smoker; glomerular filtration rate and the presence or absence of chronic kidney disease (CKD) (creatinine clearance < 60 ml/min); previous CAD (as described in electronic medical records).

Regarding the electrocardiogram, it was classified, according to the institutional report, as a normal electrocardiogram, presence of diffuse repolarization changes, the presence of changes suggestive of ischemia (T wave changes), the presence of an electrically inactive area, and ST-segment depression. Any other changes in the electrocardiogram (overloads, branch blocks, etc.) were categorized under "others".

Prognostic scores (TIMI, GRACE, and HEART) and pre-test probability calculator for CAD (Diamond Forrester) were also studied as continuous variables.

### Chest pain traits

To differentiate the type of chest pain in the emergency department, the following data were collected:

- Type of pain, as described by the CASS study:^14^1) type A - definitely anginal (has all the characteristics of angina such as retrosternal tightness, radiation to upper limbs and/or neck, worsening or triggered by effort, relief with rest and/or nitrate); 2) type B – probably anginal (has most but not all anginal characteristics); 3) type C – probably not anginal (has one or another anginal characteristic, with atypical manifestations);
- Pain triggered by effort or with onset at rest;
- Presence of associated symptoms: sweating and/or nausea/vomiting;
- The report of the pain is similar to a previous ACS episode;
- Pain in progressive worsening of frequency, intensity, or triggered by increasingly lesser efforts (crescendo).

### Statistical analysis

Continuous variables were described using the median and interquartile range, and categorical variables were presented using frequency and percentage. Comparison between groups was performed using the Chi-Square test (categorical variables) or Mann-Whitney test (continuous variables). Factors associated with outcomes were evaluated using Multiple Logistic Regression with a stepwise variable selection criterion. The level of significance considered in the analyses was 5%. The software used was "The R Foundation for Statistical Computing, version 4.2.0, 2022". The Shapiro-Wilk test was used to test the normality of continuous variables, none of which showed normal distribution.

## Results

From July 16, 2018, to February 28, 2020, 898 patients were admitted with a final diagnosis of Unstable Angina according to the ICD-10 admission code. After reviewing clinical history, electrocardiograms, and other exclusion criteria, a sample of 729 patients was obtained. There was a predominance of males (64.6%), with ages ranging from 33 to 91 years and an average of 62.9. The prevalence of major comorbidities was 82% hypertension, 46% diabetes, 58% dyslipidemia, 18% smokers, 19% with CKD, and 62% with previous CAD.

### Evaluation of the stratification method

Table 1 presents the comparison between invasive and non-invasive stratification groups, considering all analyzed variables. Of the 729 participants, 81.7% were stratified invasively (cardiac catheterization as the first examination). Of the 133 patients stratified non-invasively, 96 (72.2%) underwent myocardial perfusion scintigraphy, 31 (23.3%) underwent coronary angiography, 2 (1.5%) underwent cardiac MRI with stress, and 4 (3%) underwent exercise testing.

**Table 1:** Baseline characteristics and association with stratification method

Variable			Stratification Method		p-value
			Invasive (N=596)	Non-invasive (N=133)	
**Medical History**					
	Age, years (median, [Q1; Q3])		63 [56;69]	63 [55;71]	0.966
	Male gender		64.4%	65.4%	0.909
	BMI > 30 kg/m²		28.4%	30.8%	0.643
	SAH		82.4%	81.2%	0.845
	DM		45.6%	50.4%	0.371
	DM ID		10.9%	15.0%	0.357
	DLP		59.4%	51.9%	0.136
	CKD		18.8%	18.8%	1.000
	Active smoking		13.6%	8.27%	0.001
	Previous CAD		62.2%	60.2%	0.725
**Chest Pain**					
	Type (classification):				<0.001
		A	25.1%	11.5%	
		B	48.5%	48.1%	
		C	26.4%	40.5%	
	Rest / Effort				0.127
		Effort	34.0%	26.6%	
		Rest	66.0%	73.4%	
	'Crescendo'		26.8%	15%	0.006
**Scores**					
	GRACE (median, [Q1; Q3])		92.5 [75;110]	92 (24)	0.881
	TIMI (median, [Q1; Q3])		4 [3;4]	3 [2;4]	0.006
	HEART (median, [Q1; Q3])		5 [5;6]	5 [4;6]	0.011
**ECG normal**			108 (18.1%)	28 (21.1%)	0.508
**DVAR**			166 (27.9%)	36 (27.1%)	0.940
**EIA**			93 (15.6%)	18 (13.5%)	0.640
**Isquemia**			72 (12.1%)	9 (6.77%)	0.107
**ST Dep (mm)**					0.660
	0		584 (98.0%)	133 (100%)	
	0.5		1 (0.17%)	0 (0.00%)	
	1		9 (1.51%)	0 (0.00%)	
	2		2 (0.34%)	0 (0.00%)	

Source: prepared by the author.

BMI: body mass index; SAH: systemic arterial hypertension; DM: diabetes mellitus; DM ID: insulin-dependent diabetes mellitus; DLP: dyslipidemia; CKD: chronic kidney disease; CAD: coronary artery disease; ECG: electrocardiogram; DVAR: diffuse ventricular repolarization changes; EIA: electrically inactive area; ST Dep: ST-Segment depression.

The invasive stratification group had more smokers and former smokers (p-value = 0.001) than the non-invasive stratification group. There was no statistical difference in other baseline characteristics associated, including comorbidities such as diabetes, hypertension, or known previous CAD.

Regarding chest pain characteristics, the only variables associated with a higher chance of cardiac catheterization were type A pain (p-value < 0.001) and "crescendo" pain characteristic (p-value = 0.006). None of the other pain-related variables were statistically significant for association.

There was no statistical difference in any studied electrocardiographic variable. Changes suggestive of ischemia were found in 12.1% of patients who underwent catheterization, contrasted with 6.77% of patients with conservative stratification (p-value = 0.107). No patient with ST-segment depression on the admission electrocardiogram underwent non-invasive stratification.

The associations of risk scores with the indication for invasive stratification are also demonstrated at the end of Table 1 . Both the TIMI score (p-value = 0.006) and the HEART score (p-value = 0.011) were associated with invasive stratification. However, HEART had the same median value. The median GRACE score was 92.5 in the invasive stratification group and 91.0 in the non-invasive group. Only 3.3% (24 patients) of the entire sample had a GRACE score greater than 140, with 92% (22) of them stratified with cardiac catheterization. Twenty-six percent of patients had a GRACE score between 109 and 140, with 71% being stratified invasively.

To assess the factors that are jointly most associated with the method of stratification, all variables from the above tables were placed in a multiple logistic regression model, shown in Table 2 . The variables that best explain the likelihood of cardiac catheterization as an initial strategy are smoking and type of pain. Active smokers and former smokers were 2.23 and 2.19 times more likely, respectively, to be stratified with an invasive strategy than patients who never smoked. Patients with type A chest pain were 3.39 times more likely to undergo catheterization as initial stratification than patients with type C pain.

**Table 2:** Patient variables that individually present a higher chance of invasive stratification

Variable	Category	OR	CI 95%	p-value
Smoking	Active	2.23	1.13-4.80	0.028
	Stopped < 6m	0.84	0.41-1.85	0.646
	Former	2.19	1.39-3.53	0.001
Type of Pain	A	3.39	1.83-6.66	<0.001
	B	1.48	0.97-2.27	0.070

Source: prepared by the author.

Considering the indications of the Brazilian guideline of 2021^9^for invasive stratification ( Table 1 , chapter 1.4), 94.3% of the entire sample should have undergone invasive initial stratification. According to the European guideline of 2020,^8^15.5% of the sample should have undergone cardiac catheterization as an initial strategy.

### Evaluation of "Obstructive CAD or Ischemia" in the Population

After analyzing the results of the initial stratification exams performed on the 729 participants, 520 (71.3%) showed changes suggestive of "Obstructive CAD or Ischemia". Table 3 demonstrates the comparison between groups with and without "Obstructive CAD or Ischemia," considering all analyzed variables. Patients with "Obstructive CAD or Ischemia" had a median of two more days of hospital stay (p-value < 0.001). In univariate analysis, the group with "Obstructive CAD or Ischemia" showed a higher prevalence of male patients (p-value = 0.032). None of the other variables related to baseline characteristics showed a statistical difference. The presence of known previous CAD was slightly more prevalent in the group with "Obstructive CAD or Ischemia," but not significantly (63.5% versus 57.9%; p-value = 0.188). A higher prevalence of CKD was observed in the group with altered exams, which also did not reach statistical significance (20.4% versus 14.8%; p-value = 0.103).

**Table 3:** Baseline characteristics and association with "Obstructive CAD or Ischemia" in stratification exams

Variable			Obstructive CAD or Ischemia		p-value
			No (N = 209)	Yes (N = 520)	
**Medical History**					
	Age, years (median, [Q1; Q3])		63 [55.0;70.0]	63 [56.0;70.0]	0.399
	Male gender		58.4%	67.1%	0.032
	BMI > 30 kg/m^2^		30.1%	28.3%	0.678
	SAH		84.7%	81.2%	0.307
	DM		48.3%	45.8%	0.587
	DM ID		9.57%	12.5%	0.277
	DLP		60.8%	56.9%	0.386
	CKD		14.8%	20.4%	0.103
	Active smoker		11%	13.3%	0.439
	Previous CAD		57.9%	63.5%	0.188
**Chest Pain**					
	Type (classification)				0.105
		A	17.4%	24.7%	
		B	51.7%	47.1%	
		C	30.9%	28.2%	
	Rest / Effort				0.037
		Effort	26.7%	35.1%	
		Rest	73.3%	64.9%	
	`Crescendo’		20.6%	26.3%	0.124
**Scores**					
	GRACE (median, [Q1; Q3])		91 [74;111]	92 [75;109]	0.494
	TIMI (median, [Q1; Q3])		3 [2;4]	4 [3;4]	0.001
	HEART (median, [Q1; Q3])		5 [4;6]	5 [4;6]	0.542
**ECG normal**			16.7%	19.4%	0.463
**DVAR**			30.6%	26.5%	0.307
**EAI**			11.5%	16.7%	0.095
**Isquemia**			8.13%	12.3%	0.136
**ST Dep**					0.646
	0		99.5%	97.9%	
	0.5		0.00%	0.19%	
	1		0.48%	1.54%	
	2		0.00%	0.38%	

Source: prepared by the author.

BMI: body mass index; SAH: systemic arterial hypertension; DM: diabetes mellitus; DM ID: insulin-dependent diabetes mellitus; DLP: dyslipidemia; CKD: chronic kidney disease; CAD: coronary artery disease; ECG: electrocardiogram; DVAR: diffuse ventricular repolarization changes; EIA: electrically inactive area; ST Dep: ST-Segment Depression.

The only chest pain characteristic variable that, in univariate analysis, was significantly associated with "Obstructive CAD or Ischemia" was pain triggered by effort (p-value = 0.037). Type A chest pain (24.7% versus 17.4%; p-value = 0.105) and "crescendo" pain characteristic (26.3% versus 20.6%; p-value = 0.124) were more prevalent in the "Obstructive CAD or Ischemia" group, without reaching statistical significance.

There was no statistical difference in any studied electrocardiographic variable. Patients with positive results in stratification exams showed a tendency towards a higher prevalence of ischemia and electrically inactive areas on the ECG but without achieving statistical significance.

Regarding prognostic scores, it is noted that the group with "Obstructive CAD or Ischemia" showed a higher value in the TIMI score (median 4 versus 3; p-value = 0.001), the only prognostic score that showed a statistical difference.

To assess the factors that, together, were most associated with "Obstructive CAD or Ischemia," all variables from Table 1 were included in a multiple logistic regression model. As shown in Table 4 , the variables that, after multivariate analysis, best explain the likelihood of changes in the initial stratification exams suggestive of "Obstructive CAD or Ischemia" are the classification of chest pain and the presence of previous CAD.

**Table 4:** Patient variables that individually present a higher chance of `Obstructive CAD or Ischemia’ in stratification exams

Variable	Category	OR	CI 95%	p-value
Type of Pain	B	0.60	0.38-0.93	0.026
	C	0.61	0.37-1.00	0.051
Previous CAD	Yes	1.42	1.01-2.00	0.048
LOS		1.13	1.08-1.19	<0.001

Source: prepared by the author.

CAD: coronary artery disease; LOS: length of hospital stay.

Patients with chest pain classified as type A have a 66% (1/0.6) higher chance of CAD than patients classified as type B; patients with previous CAD have a 42% higher chance of CAD than patients without previous CAD. The presence of "Obstructive CAD or Ischemia" was also associated with a longer hospital stay in the multivariate analysis.

## Discussion

The first notable point regarding the data presented concerns the profile of patients admitted with unstable angina at the studied service. We observed an increased prevalence of comorbidities classically associated with CAD. While the FRISC II study,^6^one of the main randomized clinical trials for SCASSST stratification, showed a prevalence of 30% hypertension, 13% diabetes, and 23% with previous infarction, the population in this tertiary center had 80% hypertension, 47% diabetes, and 60% with previous CAD. This certainly does not represent the Brazilian population profile but rather the complexity of patients in a tertiary emergency department of the public health system.

Regarding baseline characteristics, it was plausible to expect that a higher prevalence of these comorbidities would assist in the indication of catheterization in the emergency scenario, as seen both by the increased probability of CAD and by the guidelines in force at the time.^10 , 11^However, the only comorbidity more prevalent in the invasive stratification group was smoking. That is, patients with some history of smoking tended to be directed towards invasive strategy upon emergency department admission.

When analyzing the prevalence of comorbidities with the presence of obstructive CAD or ischemia in the exams, the studied sample presents a peculiarity: only the male sex was independently associated. In multivariate analysis, the presence of known previous CAD, as expected and plausibly, was associated with obstructive CAD in unstable angina. The fact that hypertension, diabetes, smoking, and other comorbidities were not associated with obstructive CAD could be a bias of the studied population due to the high prevalence of these comorbidities, as mentioned earlier. In other words, the tendency to indicate catheterization for smokers was not reflected in changes in the exams.

Electrocardiographic changes suggestive of ischemia on admission ECG were few, failing to reach statistical significance in any studied data. However, relative numbers show a tendency towards a higher prevalence of electrically inactive areas and ischemic changes in patients with obstructive CAD, which may reinforce the guidelines’ recommendations to indicate cardiac catheterization in these patients. The electrocardiogram has great prognostic value, especially in the presence of ST-segment depression, which is one of the criteria of the TIMI score,^12^and was identified as a high-risk criterion in the TATICS-TIMI 18 study.^5^The guidelines^8 , 9^reinforce the indication of a direct invasive strategy in the case of this electrocardiographic alteration, and it was observed in the studied sample that 100% of the patients with ST-segment depression underwent cardiac catheterization as initial stratification.

Regarding diagnostic scores, the TIMI and HEART scores were statistically associated with the indication for invasive stratification as an initial strategy. However, the clinical relevance of this association seems to be of little significance since the difference in medians is very small (3 to 4 in TIMI and 5 to 5 in HEART, despite the difference in p-value). Furthermore, the difference identified in HEART is probably related to the type of pain, a variable included in this calculator, as in multivariate analysis, both scores do not appear indicative. The same reasoning applies to the association found of TIMI with obstructive CAD or ischemia: despite statistical significance, the clinical significance of the found difference is minimal.

The results regarding the GRACE score are very striking. According to the current European guideline,^8^we should indicate cardiac catheterization in unstable angina as an initial strategy only for patients with a GRACE score higher than 140, electrocardiographic alterations, or some degree of instability. Moreover, only 3.3% of the general population of unstable angina was at high risk by this score, demonstrating the great disparity between what is stated in the guidelines and what is performed in clinical practice. Additionally, although it has been shown to be an excellent predictor of events in the studies in which it was validated,^13^it was not associated with the presence of obstructive CAD or ischemia in the results of complementary exams.

Despite the guidelines, both Brazilian and European, suggesting a flow for the management of unstable angina, we perceive a significant difference between the guidance of the guidelines and what occurs in a reference center (tertiary) of the Brazilian public health service. While by the Brazilian guideline of 2021, 94% of the sample should have been stratified by catheterization (mostly due to the indication of catheterization in intermediate-risk patients),^9^by the European guideline of 2020,^8^only 15%. Furthermore, none of them mentions the characteristic of chest pain as a criterion for choosing the method of stratification. Moreover, in the sample of the present study, the type of pain was the most relevant variable in choosing the strategy: patients with definitely anginal pain were 3.39 times more likely to undergo cardiac catheterization and not the use of prognostic scores.

Surprisingly, the finding of the presence of previous CAD did not influence the choice of the stratification method in these patients hospitalized with UA. A possible explanation for this is due to the high prevalence of CAD in this population (62%), which may bias the findings.

When we analyze the prevalence of obstructive CAD or ischemia in men with type A pain (79%), we see an approximation with data from the CASS study,^14^which in 1989 described a 93% prevalence of obstructive CAD in men with this type of pain. pain. The CASS study excluded ACSs, but the similarity between the findings reinforces the concept of the cardiovascular "continuum", that coronary disease is one and that the differences between its stages are more prognostic than pathophysiological.

Type A chest pain (with all the characteristics suggestive of ACS) was associated with a 66% greater chance of having obstructive CAD or ischemia. While guidelines recommend invasive stratification only in patients classified as intermediate^9^or high risk,^8^guiding the type of stratification by the type of pain can optimize the management of unstable angina.

One of the main limitations of the study is the bias of the sample studied, as it is a single-center, tertiary study, of increased complexity. We should not extrapolate the findings to other populations. And, due to the type of outcome analyzed, the findings cannot be extrapolated to the view of clinical benefit, since the outcome analyzed was the change in the stratification exam and not hard outcomes such as mortality or cardiovascular events. The answer in the literature regarding the real clinical benefit of direct cardiac catheterization in unstable angina (negative troponin) still remains uncertain.

## Conclusion

The factors independently associated with the indication of invasive stratification of unstable angina in a tertiary cardiology center in the Brazilian public health system are smoking and chest pain classification. The factors independently associated with obstructive CAD or ischemia were the presence of previous CAD and chest pain classification.

Our results emphasize the importance of chest pain assessment not only for the diagnosis of unstable angina but also in its use for defining the method of stratification.
